# The AACE 2022 Guideline: An Academic Appraisal

**DOI:** 10.17925/EE.2023.19.1.2

**Published:** 2023-03-31

**Authors:** Saptarshi Bhattacharya, Sanjay Kalra

**Affiliations:** 1. Department of Endocrinology, Indraprastha Apollo Hospitals, New Delhi, India; 2. Department of Endocrinology, Bharti Hospital, Karnal, India; 3. University Center for Research & Development, Chandigarh University, Chandigarh, India

**Keywords:** Diabetes, diabetes complications, person-centred care, prediabetes, type 2 diabetes

## Abstract

The American Association of Clinical Endocrinology (AACE) 2022 guideline provides comprehensive and evidence-based guidance on contemporary diabetes management. The statement reiterates the importance of person-centred, team-based care for optimum outcomes. The recent strides to prevent cardiovascular and renal complications have been aptly incorporated. The recommendations on virtual care, continuous glucose monitors, cancer screening, infertility and mental health are relevant. However, focused discussions on non-alcoholic fatty liver disease and geriatric diabetes care could have been helpful. Outlining targets for prediabetes care is a notable addition and is likely to be the most effective strategy in addressing the rising burden of diabetes.

The American Association of Clinical Endocrinology (AACE) recently released a comprehensive and updated guideline on the prevention and management of diabetes.^1^ The guideline is aimed at assisting healthcare professionals in delivering person-centred optimal care at different phases of diabetes. The statement comprises 170 evidence-based recommendations divided into four sections.

## Noteworthy additions

There are several new and noteworthy updates in the AACE 2022 guideline. An elaborate section on inpatient hyperglycaemia, where issues related to coronavirus disease 2019 (COVID-19) have also been discussed, is a valuable inclusion. The importance of age-appropriate vaccination, including the COVID-19 vaccine, in people with diabetes has been rightly emphasized. Delivery of telehealth and virtual care platforms has been reviewed, conforming to the changing trends. Continuous glucose monitoring is another technological advancement finding widespread application in current diabetes management. The guideline aptly recognizes the need for incorporating information and insights from continuous glucose monitoring, especially for patients on insulin.

Specific recommendations on approaches to male and female infertility, post-transplant and secondary diabetes are relevant. The guideline also underlines the close link between cancer and diabetes, and highlights the requirement for necessary screening strategies. The task force has rightfully focused on the impact of quality of life, sleep, depression and social determinants of health on outcomes. However, attention to certain aspects will enhance the global reach of the document.

## Screening, diagnosis and monitoring

The first section of the guideline addresses screening, diagnosis, glycaemic targets and monitoring. An effective screening policy is critical to tackling the global burden of diabetes and associated complications. The AACE guideline recommends periodic screening for diabetes after the age of 35 years.^1^ Given the significant racial differences in predisposition to diabetes, Asian, Hispanic and African individuals should be screened at a younger age.^2^ Specific age cut-offs for screening in high-risk ethnic groups could be a cost-effective strategy.^3^

The task force recommends screening all pregnant women during the first prenatal visit to identify undiagnosed diabetes. In the process, a significant proportion of women is found to have moderate hyperglycaemia, which falls short of the levels needed for diagnosing overt diabetes, but fulfils the conventional criteria for diagnosing gestational diabetes mellitus (GDM). This common condition, often labelled as early GDM, lacks a defined treatment strategy.^4^ The AACE 2022 guideline remains silent on diagnosing and treating early GDM. The American Diabetes Association (ADA) 2022 statement, however, does recognize the condition and acknowledges the uncertainty around its diagnosis and management.^5^

## Comorbidities and complications

The next section of the AACE guideline focuses on personalized management of comorbidities, such as hypertension and dyslipidaemia, and complications of diabetes. Both the AACE 2022 and ADA 2023 guidelines recommend blood pressure targets <130/80 mmHg for patients with hypertension and diabetes. The AACE guideline further suggests that a lower blood pressure target of <120/70 mmHg can be considered in those at high-risk of cardiovascular disease. The recommendation is based on the findings of the Systolic blood pressure intervention trial (SPRINT), a study that excluded patients with diabetes.^6^ On the other hand, the trial involving participants with diabetes only, Action to control cardiovascular risk in diabetes blood pressure (ACCORD BP), did not confirm the benefits of a lower target.^7^ The risks versus benefits of aggressive blood pressure lowering to <120/70 mmHg, as suggested in the AACE guideline, have to be balanced, and a more conventional target of <130/80 mmHg may be prudent until we have evidence to justify the lower target.

The guideline also advocates using fibrates if triglycerides levels are <200 mg/dL and lipids are not on target after maximum tolerated statin, to achieve an apolipoprotein-B target of <90 mg/dL. Although there is evidence for the use of apolipoprotein-B as a marker of residual cardiovascular risk, its routine use as a monitoring tool can be challenging in resource-l imited settings.^8,9^ The common complications of diabetes have been addressed in the guideline; however, a dedicated discussion on non-alcoholic fatty liver disease could have been helpful.

## Management of prediabetes and diabetes

The third section comprehensively discusses management of prediabetes, type 1 and type 2 diabetes, GDM and in-hospital hyperglycaemia. Outlining the targets for prediabetes management is a novel addition. The discussion on the role of obesity medicines in prediabetes is appropriate. The task force has preferred the term adiposity-based chronic disease in place of obesity in the statement, as this term reinforces obesity as a chronic condition that requires specialized management, alludes to the pathophysiology and also lessens the associated stigma attached to the term.^10^ On the same lines, a discussion on the importance of communication in diabetes could have been considered.

The recommendations on in-hospital management of hyperglycaemia are relevant. Glucocorticoid use and infection are the two major reasons behind worsening of glycaemic control in hospital settings, and might have warranted a focused discussion.

## Additional aspects of diabetes

The final section discusses several important aspects such as education, infertility, sleep, mental health, virtual health, occupational safety, nutritional supplements and vaccination. Male hypogonadism is a common but often overlooked complication of diabetes, and it is appropriately discussed in the guidelines. The importance of depression and diabetes distress has been highlighted, and the need for routinely evaluating mental health in people with diabetes has been emphasized.

A discussion on the non-pharmacological components of a care plan for children and adolescents is appreciated. There is also scope for a segment on pharmacological and non-pharmacological management of diabetes in geriatric patients. Also, considering the rapid rise in global numbers of people with diabetes, incorporating positive aspects of non-conventional therapies, such as yoga, mindfulness, meditation and integrative medicine, will be a welcome step to cope with the challenge of the diabetes pandemic.^11–14^

## Conclusion

The AACE guideline is a well-written, comprehensive, evidence-based opinion piece about the prevention and management of diabetes. While we understand the document is predominantly targeted at a US audience, a few additions will increase its global relevance. This will bring uniformity to the care of diabetes worldwide. A discussion on newer drugs, some of which are not available in the USA, and non-pharmacological measures commonly practiced in many parts of the world will make the document more inclusive. Few areas of medicine require as much individualization, person-centred approaches and self-engagement as diabetes. The AACE 2022 guideline successfully incorporates the dynamicity of diabetes management in its recommendations. It is heartening to see the evolution of the document over time, from a scientific and humane perspective.
